# Intra- and Interrater Reliability of Short-Term Measurement of Heart Rate Variability on Rest in Individuals Post-COVID-19

**DOI:** 10.3390/ijerph192013587

**Published:** 2022-10-20

**Authors:** Lucivalda Viegas de Almeida, Aldair Darlan Santos-de-Araújo, Rodrigo Costa Cutrim, Rudys Rodolfo de Jesus Tavarez, Audrey Borghi-Silva, Fábio Henrique Ferreira Pereira, André Pontes-Silva, Adriana Sousa Rêgo, Daniel Santos Rocha, Renan Shida Marinho, Almir Vieira Dibai-Filho, Daniela Bassi-Dibai

**Affiliations:** 1 Postgraduate Program in Programs Management and Health Services, Universidade Ceuma, São Luís 65075-120, MA, Brazil; 2 Grupo de Pesquisa em Avaliação e Reabilitação Cardiovascular, Respiratória e Metabólica, Universidade Ceuma, São Luís 65075-120, MA, Brazil; 3 Department of Physical Therapy, Universidade Federal de São Carlos, São Carlos 13565-905, SP, Brazil; 4 Cardiopulmonary Physiotherapy Laboratory—LACAP, Universidade Federal de São Carlos, São Carlos 13565-905, SP, Brazil; 5 Postgraduate Program in Dentistry, Universidade Ceuma, São Luís 65075-120, MA, Brazil; 6 Postgraduate Program in Environment, Universidade Ceuma, São Luís 65075-120, MA, Brazil; 7 Department of Physical Therapy, Universidade Ceuma, São Luís 65075-120, MA, Brazil; 8 Postgraduate Program in Adult Health, Universidade Federal do Maranhão, São Luís 65080-805, MA, Brazil; 9 Grupo de Pesquisa em Reabilitação, Exercício e Movimento (REMOVI) Universidade Federal do Maranhão, São Luís 65080-805, MA, Brazil

**Keywords:** COVID-19, heart rate variability, reliability, reproducibility, Kubios Software

## Abstract

Individuals affected by COVID-19 have an alteration in autonomic balance, associated with impaired cardiac parasympathetic modulation and, consequently, a decrease in heart rate variability (HRV). This study examines the inter- and intrarater reliability of HRV) parameters derived from short-term recordings in individuals post-COVID. Sixty-nine participants of both genders post-COVID were included. The RR interval, the time elapsed between two successive R-waves of the QRS signal on the electrocardiogram (RRi), were recorded during a 10 min period in a supine position using a portable heart rate monitor (Polar^®^ V800 model). The data were transferred into Kubios^®^ HRV standard analysis software and analyzed within the stable sessions containing 256 sequential RRi. The intraclass correlation coefficient (ICC) ranged from 0.920 to 1.000 according to the intrarater analysis by Researcher 01 and 0.959 to 0.999 according to the intrarater by Researcher 02. The interrater ICC ranged from 0.912 to 0.998. The coefficient of variation was up to 9.23 for Researcher 01 intrarater analysis, 6.96 for Researcher 02 intrarater analysis and 8.83 for interrater analysis. The measurement of HRV in post-COVID-19 individuals is reliable and presents a small amount of error inherent to the method, supporting its use in the clinical environment and in scientific research.

## 1. Introduction

In addition to directly infecting cells of the respiratory tract, the inflammatory response from SARS-CoV-2 infection can lead to systemic inflammation and affect almost all organ systems, including the cardiovascular system and the autonomic nervous system (ANS). Damage to these systems is associated with multiple factors and may result from an imbalance between high metabolic demand, low cardiac reserve, thromboembolism, and direct tissue damage caused by the virus [1,2,3]. Physiologically, the persistent inflammatory process in this population is mediated by the ANS through a rapid reflex action involving a central set of structures, especially the vagus nerve, which is considered the primary interface between inflammation and action in the central nervous system, in addition to being an important neuroimmunomodulator in the adaptive regulation of the inflammatory process [4,5].

Considering the close relationship between the vagus nerve and cardiac autonomic control, the heart rate variability (HRV), a simple, non-invasive, objective and validated physiological metric for the assessment of the autonomic nervous system, has been used in the most diverse scientific and clinical contexts, whether for diagnostic or prognostic purposes [4,6]. As the ANS is sensitive to physiological changes and capable of quickly generating adaptive responses, it can offer signals, in the form of an HRV, that can quickly warn of an imminent health risk [5]. Recent studies have shown that individuals affected by COVID-19 have an alteration in autonomic balance, associated with impaired cardiac parasympathetic modulation and, consequently, a decrease in HRV, which may persist for at least six months after hospital discharge [7,8,9]. Furthermore, in those individuals who required hospitalization, a lower HRV was associated with the prediction of admission to the intensive care unit in the first week of infection, whereas those who had a higher HRV had a higher chance of survival [10].

Methodologically, the acquisition of the biological signal for the processing of HRV can be done through non-invasive tools, such as an electrocardiogram or a heart rate monitor [6]. Although the acquisition of metrics related to heart rate and HRV are obtained with greater precision using an electrocardiogram of multiple variations, the use of this type of equipment is unfeasible due to its availability in most cases only in large research centers and hospitals. There is a high level of knowledge required for data collection and processing, which leads the industry to produce and expand valid handheld devices that are easy to manage, collect and analyze [11]. Despite the large scientific collection available on the subject, the processing and analysis of the HRV signal continues to be a challenge for researchers. This is because the choice of the best stationary stretch, in addition to being manually and visually selected, depends on the subjective decisions of the evaluator. This can generate biases in the results and differences between and among researchers capable of underestimating or overestimating the values found and influencing the clinical interpretation [6,12,13,14].

Although the measurement of HRV can easily become subject to bias in the face of inadequate collection and equivocal analysis, few studies have been directed to measure its reliability [15,16,17]. Above all, in individuals who are victims of COVID-19, where the severity of the disease can influence the levels of inflammatory cytokines and impair the autonomic and cardiovascular nervous system, the evaluation of the HRV measurement provides subsidies that can influence the decision-making process evaluation and treatment decisions. However, the technical aspects involving the collection and analysis can affect the reproducibility of this measure and generate dubious results and considerable clinical impacts [18,19].

Considering this context and the growing investigations involving HRV in individuals affected by COVID-19, this study aims to evaluate the intra- and interrater reliability of the short-term HRV measurement in post-COVID-19 individuals. The main contributions of this research are: 1. Even though the best stationary parts of the HRV are selected for analysis, they are evaluator-dependent, that is, prone to errors; 2. Researchers who analyze HRV signals must have previous experience and training, to reduce the chance of bias in the analysis; 3. In post-COVID-19 patients, excellent intra- and inter-researcher reliability values were found, supporting its clinical applicability

## 2. Materials and Methods

### 2.1. Study Design

**Figure 1:**
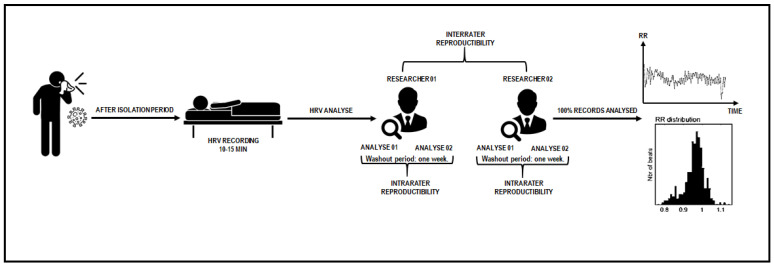
Study design illustration.

This is a reliability study conducted according to the Guidelines for Reporting Reliability and Agreement Studies (GRRAS) [20]. The research was conducted at the Universidade Ceuma (São Luís, MA, Brazil) after the study procedures were approved by the Research Ethics Committee of the institution (protocol number: 4.179.747) and conducted according to the Declaration of Helsinki. All participants were informed of the purpose of the study and informed consent was obtained. Study design is graphically presented in Figure 1.

### 2.2. Participants

Individuals of both sexes aged ≥18 years, positive for SARS-CoV-2 according to the RT-PCR test, were included. The stratification followed the following classifications according to the COVID-19 Treatment Guidelines [21] produced by the National Institute of Health: mild cases (individuals who presented signs and symptoms of the disease, such as fever, cough, sore throat, malaise, headache pain, muscle pain, nausea, vomiting, diarrhea, loss of taste and smell, but without the need for additional oxygen therapy and/or mechanical ventilation); moderate cases (individuals who showed evidence of respiratory disease or imaging with oxygen saturation ≥ 94%); severe cases (individuals with SpO_2_ < 94% on room air, with a ratio of arterial oxygen partial pressure to inspired oxygen fraction (PaO_2_/FiO_2_) < 300 mmHg, respiratory rate > 30 breaths/min, or lung infiltrates > 50%); critical cases (individuals who have respiratory failure, need for high-flow oxygen therapy, invasive or non-invasive mechanical ventilation).

Volunteers who had myocardial infarction (within six months from the start of data collection), an implanted pacemaker or any metallic synthesis, a history of heart disease, unstable angina, uncontrolled hypertension, uncontrolled diabetes mellitus and/or insulin-dependent, chronic obstructive pulmonary disease, neoplasms, cognitive deficit, declared users of illicit drugs, and pregnant were not included in the study.

The sample size calculation was performed according to the study carried out by Fleiss. We considered the following criteria for the calculation: minimum acceptable ICC value of 0.40, expected ICC of 0.75 (moderate reliability), alpha error of 5%, power of 80%, sample loss rate of 15%. Therefore, a minimum sample size of 33 volunteers was estimated.

### 2.3. Habitual Physical Activity

The Baecke Habitual Physical Activity Questionnaire is a self-applicable, self-report instrument that assesses physical activity over the past 12 months. It consists of 16 items, divided into 3 domains: physical activity in occupation (items 1–8), physical activity in sports in free time (items 9–12), and physical activity during leisure other than sport (items 13–16). There are five Likert scale (1–5) response possibilities. For each domain, the final score ranges from 1 to 5; the higher the score, the higher the level of physical activity [22,23].

### 2.4. Heart Rate Variability Assessment

Volunteers were positioned supine in a calm environment with controlled temperature between 22–24 °C and relative humidity between 50–60% in the morning and were encouraged to remain relaxed, breathe normally, not talk, fidget or sleep during the session. They were instructed not to ingest any stimulant substance, not to perform any strenuous exercise, and to have a good night’s sleep the day before and the day of the exam. Before the collection, the volunteers remained relaxed for 10 min to stabilize the heart rate and after this period of time the collection started [6].

### 2.5. Heart Rate Collection and RR Intervals

Heart rate and RR intervals (iR-R) (time interval between complex peaks (QRS) which are determined by modulation of the sympathetic and parasympathetic nervous system, were obtained by a heart rate monitor (Polar V800, Polar Electro Inc., Bethpage, NY, USA), sampling frequency of 1000 Hz, recorded continuously for a period of 10 min with a chest strap and the volunteer in the supine position. Data were saved as iR-R data files, with intervals in ms. The data were exported from the Polar Flow web service as a space delimited .txt file [24].

### 2.6. Data Processing and Analysis

Data were transferred to Kubios^®^ HRV Standard analysis software (MATLAB, version 3.5, Kuopio, Finland). O software Kubios is an easy-to-use HRV analysis tool including a wide variety of time-domain, frequency-domain and nonlinear analysis options. Another important fact is that the software is available free of charge for Windows, Linux and iOS operating systems [14].

After transferred to Kubios^®^ the data were analyzed within stable sessions containing 256 sequential RRi. Initially, a visual inspection of the collected signals was performed. If there were 10% of ectopic beats in relation to the total pure sinus beat, the data were discarded. For the selection of the best stationary period, the following criteria were considered: (1) no large R–R interval outliers (i.e., a R–R interval much higher or lower than the whole R–R signal, based on the visual inspection of the HRV recording performed by the researcher), (2) R–R intervals equidistance and (3) Gaussian R–R intervals and heart rate distribution graphics [17].

Following the guidelines of the Task Force of the European Society of Cardiology [13], we derived variables from linear analysis (time and frequency-domain) and nonlinear analysis. For time-domain analysis, the following variables were analyzed: the mean of intervals RR in milliseconds (ms) (Mean RR), the standard deviation of all normal R-R intervals (SDNN) in ms, the mean of heart rate in beats per minute (bpm) (Mean HR), the squared root of the mean of the sum of the squares of successive normal R–R interval differences (RMSSD) in ms and the integral of the density of the RR interval histogram divided by its height (RR Tri). For frequency-domain the following variables were analyzed: low-frequency band (0.04–0.15 Hz) in normal units and milliseconds (L.F.) and high-frequency band in normal units and milliseconds (0.15–0.4 Hz) (H.F.) [25].

Finally, for non-linear analysis, the following variables were used: approximate entropy (ApEn) and sample entropy (SampEn), which measures the regularity and complexity of a time series and were proposed to quantify the entropy rate of short to medium-length NN series, detrended fluctuation analysis, which describes short-term fluctuations (DFA α1) and detrended fluctuation analysis, which describes long-term fluctuations (DFA α2) [25].

### 2.7. Statistical Analysis

The intra- and interrater reliability of HRV parameters was analyzed using the following statistical procedures: intraclass correlation coefficient (ICC)_2,1_ [26], confidence interval (95% IC), standard error of measurement (SEM) and minimum detectable change (MDC), coefficient of variation (CV) and Bland–Altman plots (mean difference [bias] and limits of agreement). The interpretation of the ICC_2,1_ value was based on the study by Fleiss [27]: for values below 0.40, reliability was considered low; between 0.40 and 0.75, moderate; between 0.75 and 0.90, substantial; and finally, for values greater than 0.90, reliability was rated as excellent.

SEM, MDC and CV values were added in order to complement the interpretation of the error of the HRV measurement method. SEM can be defined as an estimate of the expected random variation in scores when no real change has taken place and was calculated using the formula: standard deviation of means×√(1−ICC) [14,28]. MDC can be defined as the minimal change that falls outside the measurement error in the result of an instrument used to measure a clinical characteristic and was calculated using the formula: $1.96\times SEM\times \sqrt{2}$ [14,28]. The CV is the ratio of the standard deviation to the mean value and represents the extent of variability of an assay. It is expressed as a percentage of deviation from the mean; the larger the CV, the greater the error in the assay. The formula for computation of CV is straightforward: CV(%) = $\left(\frac{standard\;deviation}{mean}\right)\times 100$ [29]. The Bland Altman Plots is an alternative analysis, based on the quantification of the agreement between two quantitative measurements by studying the mean difference and constructing limits of agreement. The midline represents the mean systematic difference between inter and intrarater scores ($\bar{d}$) and can be interpreted as being bias estimated by the mean difference between the two measures (X1 − X2)/n, where “n” represents the number of individuals included in the sample. The two dotted lines above and below the line mean represent the limits of agreement, and these are drawn at $\bar{d}$ ± 1.96 × s, where “s” represents standard deviation of the differences [30,31]. All analyses were performed using the Statistical Package for the Social Sciences software (SPSS version 20, SPSS Inc., Chicago, IL, USA).

## 3. Results

Table 1 describes personal, clinical and anthropometric data. We included 69 participants with a confirmed diagnosis of post-COVID-19 in this study. Of these, 43 (62.3%) were women and 26 (37.7%) were men.

The mean and dispersion values of linear and nonlinear HRV indices in the supine position evaluated by Researcher 01 and Researcher 02 are described in Table 2.

Regarding the intrarater reliability of Researcher 01, in Table 3, the ICCs of HRV parameters in time-domain ranged from 0.992 to 0.999, in frequency-domain ranged 0.951 to 0.986, and in the nonlinear methods ranged 0.920 to 0.972. The SEM ranged from 0 to 4.29% in the time-domain, 7.93 to 15.04% in the frequency-domain, and 2.54 to 16.76% in the nonlinear methods. The MDC ranged from 0 to 11.90% in the time-domain, 21.99 to 41.68 in the frequency-domain, and 7.04 to 46.46% in the nonlinear methods.

Table 4 shows the results of the reliability of Researcher 02. The ICCs in time-domain ranged from 0.992 to 0.999, in frequency-domain ranged 0.987 to 0.998, and in the nonlinear methods ranged 0.965 to 0.990. The SEM ranged from 1.90 to 4.18% in the time-domain, 1.84 to 12.95% in the frequency-domain, and 1.28 to 12.04% in the nonlinear methods. The MDC ranged from 5.26 to 11.60% in the time-domain, 5.10 to 35.90% in the frequency-domain, and 3.54 to 33.37% in the nonlinear methods.

Overall, the CV for the HRV parameters ranged from 0.11 to 4.46% in the time-domain, 7.66 to 15.92% in the frequency-domain, and 1.43 to 9.23% in the nonlinear methods of intrarater reliability by Researcher 01 (Table 3); 0.76 to 3.05% in the time-domain, 3.08 to 4.60% in the frequency-domain, and 0.86 to 6.86% in the nonlinear methods in intrarater reliability by Researcher 02 (Table 4); and 0.86 to 3.97 in the time-domain, 6.02 to 14.38% in the frequency-domain, and 1.45 to 8.83% in the nonlinear methods in interrater reliability (Table 5).

**Figure 2:**
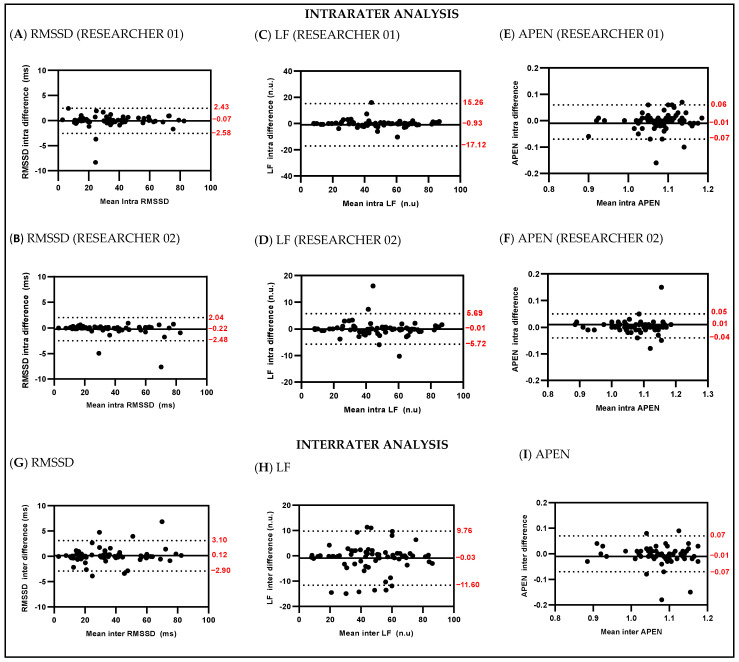
Bland-Altman plots for the RMSSD, LF and APEN intrarater and interrater.

Figure 2 shows Bland-Altman plots for RMSSD, HF and Apen evaluated by intra- and interrater. Mean inter-differences and limits of agreement (95%) for RMSSD were −0.07 (−2.58 to 2.43) by researcher 01 and −0.22 (−2.48 to 2.04) by researcher 02; for LF, −0.93 (−17.12 to 15.26) by researcher 01 and −0.01 (−5.72 to 5.69) by researcher 02; for Apen, −0.01 (−0.07 to 0.06) by researcher 01 and 0.01 (−0.04 to 0.05) by researcher 02. The interrater values for RMSSD, LF and Apen were 0.12 (−2.90 to 3.10), −0.93 (−11.60 to 9.76) and −0.01 (−0.04 to 0.05), respectively. Bland-Altman plots for all variables are presented as Supplementary Material.

## 4. Discussion

The main findings of this study are: (1) the results on the influence of selection of the best stationary stretch may reflect on the results of HRV indices when evaluated by the same researcher and by different researchers at different times in post-COVID individuals; (2) intra- and inter-researcher analyzes show excellent reliability values in patients post-COVID-19 represented by ICC values greater than 0.90; (3) the analysis variation coefficients were less than 10% in the intrarater analysis and less than 9% in the interrrater analysis; (4) the Bland–Altman plots showed wider limits of agreement in the inter-rater HRV signal data processing in comparison with the intrarater.

First, it is important to clarify that the reliability of the Polar V800 heart rate monitor has already been evaluated and validated [24,32]. However, the biggest issue would be the reliability of the analysis of these signals referring to HRV, especially in the post-COVID-19 population.

A recent systematic review investigated studies that evaluated autonomic dysfunction in patients with COVID-19 and identified seven studies that used HRV as an instrument to assess dysautonomia [7]. However, to the best of our knowledge, there is no study in the literature that has investigated the amount of error related to HRV measurement in patients with COVID-19 or post-COVID-19. Thus, our results stand out for providing clinical support for the use of HRV in these individuals, given that the error inherent in an assessment instrument is population-dependent [20].

The results of the present study are similar to research that investigated the reliability of HRV in other populations. Bassi et al. [15] identified ICC values ranging from 0.73 to 0.99 in the analysis of the intra- and inter-rater reliability of the HRV at rest in diabetic type 2 patients; Zizzo et al. [33] observed values ≥ 0.80 in the measurement of fetal HRV; and Sima et al. [34] identified ICC values ranging from 0.72 to 0.93 in chronic obstructive pulmonary disease patients.

Our study has limitations that must be considered. We included post-COVID-19 individuals, and the reliability of patients with ongoing infection was not evaluated. Our study investigated the reliability of a heart rate monitor. Thus, we recommend that future studies measure the reliability of other HRV capture devices. The reliability presented in this study is based on the HRV analysis method (comprising the identification of the best stationary stretch of the signal and measurement of parameters), according to a previously published study [15] we recommended that future studies consider measuring the reliability of the entire evaluation procedure, including patient positioning, orientations, placement of the strap or signal capture electrodes and capture of the signal.

## 5. Conclusions

This study provides a quantification of possible differences between researchers and intra-researchers, such as the influence of the selection of the iR-R by different researchers (interrater) and by the same researcher at different times in the quantification of HRV parameters (intrarater). This is extremely relevant in the context of qualitative biological variables. In this sense, the measurement of HRV in post-COVID-19 individuals is reliable and presents a small amount of error inherent to the method, supporting its use in the clinical environment and in scientific research.

## Figures and Tables

**Table 1:** Personal and clinical characteristics of the study sample.

Variables	Mean (Standard Deviation) or n (%)
Age (years)	34.20 (8.05)
Sex	
Male	26 (37.7%)
Female	43 (62.3%)
Body Mass (kg)	73.45 (22.33)
Height (m)	1.65 (0.11)
Body mass index (kg/m^2^)	26.07 (8.08)
Habitual physical activity (score, 1–15)	7.40 (1.18)
Infection time (months)	25.85 (17.91)
Severity	
Mild	53
Moderate	16
Comorbidities	
Systemic Arterial Hypertension	8 (11.60%)
Diabetes	10 (14.49%)
Obesity	5 (7.24%)
Heart diseases	2 (2.89%)

**Table 2:** Mean values and standard deviation of HRV in post-COVID-19 individuals in the supine position.

HRV Indices	Researcher 1	Researcher 1	Researcher 2	Researcher 2
	Test	Retest	Test	Restest
**TIME-DOMAIN**				
Mean RR (ms)	808.55 ± 106.85	808.33 ± 106.74	807.53 ± 106.92	808.40 ± 106.93
SDNN (ms)	31.15 ± 15.65	31.15 ± 15.73	31.10 ± 15.47	31.24 ± 15.60
Mean HR (bpm)	75.56 ± 10.61	75.58 ± 10.61	75.67 ± 10.64	75.58 ± 10.63
RMSSD (ms)	33.48 ± 20.17	33.55 ± 20.14	33.35 ± 19.94	33.57 ± 20.21
RR Tri	8.45 ± 4.04	8.34 ± 4.02	8.35 ± 3.90	8.47 ± 3.97
**FREQUENCY-DOMAIN**				
LF (n.u.)	46.51 ± 19.75	47.44 ± 18.56	47.44 ± 19.53	47.46 ± 19.66
HF (n.u.)	53.40 ± 19.77	52.49 ± 18.58	52.47 ± 19.54	52.47 ± 19.67
LF (ms^2^)	431.00 ± 428.30	453.50 ± 441.00	454.46 ± 468.01	460.05 ± 468.25
HF (ms^2^)	631.30 ± 753.40	643.70 ± 762.20	631.38 ± 769.00	640.24 ± 767.95
**NONLINEAR METHODS**				
Apen	1.08 ± 0.66	1.08 ± 0.62	1.08 ± 0.69	1.08 ± 0.70
SampEn	1.78 ± 0.25	1.78 ± 0.22	1.76 ± 0.22	1.76 ± 0.23
DFA α1	0.94 ± 0.26	0.94 ± 0.26	0.95 ± 0.26	0.95 ± 0.27
DFA α2	0.35 ± 0.15	0.37 ± 0.15	0.35 ± 0.16	0.36 ± 0.16

HRV: heart rate variability; RR: interbeat intervals between all successive heartbeats; SDNN: standard deviation of the N-N interval; RMSSD: root mean square differences of successive RR intervals; RR Tri: integral of the RR intervals histogram divided by the height of the histogram; TINN: baseline width of the RR intervals histogram; LF: normalized unit in the low-frequency band; HF: normalized unit in the high-frequency band; LF/HF: ratio of LF-to-HF; ApEn: approximate entropy; SampEn: sample entropy; DFA α1: detrended fluctuations analysis, which describes short-term fluctuations; DFA α2: detrended fluctuation analysis, which describes long-term fluctuations; ms: milliseconds; n.u.: normal units; ms: milliseconds. No significant differences were found intra or inter examinator.

**Table 3:** Intrarater reliability of HRV analysis of post-COVID-19 individuals in the supine position (Researcher 01).

HRV Index	ICC	CI 95%	SEM	SEM (%)	MDC	MDC (%)	Bias ± SD	CV (%)	95% Limit of Agreement
**TIME-DOMAIN**									
Mean RR (ms)	0.999	0.999, 1.000	0.63	1.90	1.76	5.26	0.22 ± 2.00	0.11	−3.70, 4.15
SDNN	0.998	0.996, 0.999	0.70	2.25	1.94	6.24	−0.01 ± 1.53	2.47	−3.00, 3.00
Mean HR (bpm)	0.999	0.999, 1.000	0.59	1.81	1.64	5.01	−0.01 ± 0.21	0.11	−0.42, 0.39
RMSSD (ms)	0.999	0.998, 0.999	0.64	2.28	1.77	6.31	−0.07 ± 1.28	1.87	−2.58, 2.43
RR Tri	0.992	0.986, 0.995	0.36	4.29	1.00	11.90	0.11 ± 0.73	4.46	−1.33, 1.55
**FREQUENCY-DOMAIN**									
LF (n.u.)	0.951	0.922, 0.970	4.24	9.03	11.75	25.02	−0.93 ± 8.26	7.66	−17.12, 15.26
HF (n.u.)	0.952	0.922, 0.970	4.20	7.93	11.64	21.99	0.92 ± 8.25	6.51	−15.25, 17.09
LF (ms^2^)	0.986	0.976, 0.992	62.69	14.17	173.76	39.29	−22.54 ± 305.20	15.92	−620.70, 575.70
HF (ms^2^)	0.984	0.974, 0.990	95.85	15.04	265.70	41.68	−12.36 ± 91.90	7.78	−388.40, 363.70
**NONLINEAR METHODS**									
Apen	0.920	0.871, 0.950	0.18	16.76	0.50	46.46	−0.01 ± 0.03	1.43	−0.07, 0.06
SampEn	0.963	0.941, 0.977	0.05	2.54	0.13	7.04	−0.01 ± 0.08	2.28	−0.18, 0.17
DFA α1	0.972	0.955, 0.983	0.04	4.63	0.12	12.83	0.01 ± 0.09	4.93	−0.17, 0.17
DFA α2	0.958	0.932, 0.974	0.03	8.54	0.09	23.67	−0.01 ± 0.06	9.23	−0.13, 0.10

HRV: Heart Rate Variability; RR: interbeat intervals between all successive heartbeats; SDNN: standard deviation of the N-N interval; RMSSD: root mean square differences of successive RR intervals; RR Tri: integral of the RR intervals histogram divided by the height of the histogram; TINN: baseline width of the RR intervals histogram; LF: normalized unit in the low-frequency band; HF: normalized unit in the high-frequency band; LF/HF: ratio of LF-to-HF; ApEn: approximate entropy; SampEn: sample entropy; DFA α1: detrended fluctuations analysis, which describes short-term fluctuations; DFA α2: detrended fluctuation analysis, which describes long-term fluctuations; ms: milliseconds; n.u.: normal units; ms: milliseconds.

**Table 4:** Intrarater reliability of HRV analysis of post-COVID-19 individuals in the supine position (Researcher 02).

HRV Index	ICC	CI 95%	SEM	SEM (%)	MDC	MDC (%)	Bias ± SD	CV (%)	95% Limit of Agreement
**TIME-DOMAIN**									
Mean RR (ms)	0.964	0.942, 0.978	20.29	2.51	56.23	6.96	−0.87 ± 4.72	0.76	−10.14, 8.39
SDNN	0.998	0.996, 0.998	0.69	2.23	1.93	6.18	−0.14 ± 1.10	1.29	−2.30, 2.00
Mean HR (bpm)	0.959	0.934, 0.975	2.15	2.85	5.97	7.89	0.08 ± 0.47	0.76	−0.84, 1.01
RMSSD (ms)	0.999	0.998, 0.999	0.63	1.90	1.76	5.26	−0.22 ± 1.15	1.07	−2.48, 2.04
RR Tri	0.992	0.987, 0.995	0.35	4.18	0.98	11.60	−0.12 ± 0.65	3.05	−1.41, 1.16
**FREQUENCY-DOMAIN**									
LF (n.u.)	0.988	0.980, 0.992	0.87	1.84	2.42	5.10	−0.01 ± 2.91	3.08	−5.72, 5.69
HF (n.u.)	0.987	0.980, 0.992	2.24	4.26	6.20	11.81	0.01 ± 2.91	3.10	−5.71, 5.71
LF (ms^2^)	0.984	0.974, 0.990	59.21	12.95	164.13	35.90	−5.49 ± 119.3	4.60	−239.4, 228.4
HF (ms^2^)	0.997	0.996, 0.998	42.09	6.62	116.67	18.35	−8.96 ± 80.69	3.58	−167.1, 149.2
**NONLINEAR METHODS**									
Apen	0.965	0.944, 0.978	0.13	12.04	0.36	33.37	0.01 ± 0.02	0.86	−0.04, 0.05
SampEn	0.990	0.984, 0.994	0.02	1.28	0.06	3.54	−0.01 ± 0.04	1.16	−0.08, 0.07
DFA α1	0.968	0.948, 0.980	0.05	4.99	0.13	13.83	−0.01 ± 0.07	4.66	−0.15, 0.14
DFA α2	0.969	0.950, 0.981	0.03	7.94	0.08	22.00	−0.01 ± 0.03	6.86	−0.06, 0.06

HRV: Heart Rate Variability; RR: interbeat intervals between all successive heartbeats; SDNN: standard deviation of the N-N interval; RMSSD: root mean square differences of successive RR intervals; RR Tri: integral of the RR intervals histogram divided by the height of the histogram; TINN: baseline width of the RR intervals histogram; LF: normalized unit in the low-frequency band; HF: normalized unit in the high-frequency band; LF/HF: ratio of LF-to-HF; ApEn: approximate entropy; SampEn: sample entropy; DFA α1: detrended fluctuations analysis, which describes short-term fluctuations; DFA α2: detrended fluctuation analysis, which describes long-term fluctuations; ms: milliseconds; n.u.: normal units; ms: milliseconds. Regarding interrater reliability, in Table 5, the ICCs ranged 0.958 to 0.998 in the time-domain, 0.970 to 0.979 in the frequency-domain, and 0.912 to 0.970 in the nonlinear methods. The SEM ranged from 2.54 to 4.96% in the time-domain, 6.43 to 26.59% in the frequency-domain, and 2.50 to 18.54% in the nonlinear methods. The MDC ranged from 7.05 to 13.74% in the time-domain, 17.83 to 73.70% in the frequency-domain, and 6.92 to 51.39% in the nonlinear methods.

**Table 5:** Interrater reliability of HRV analysis of post-COVID-19 individuals in the supine position.

HRV Index	ICC	CI 95%	SEM	SEM (%)	MDC	MDC (%)	Bias ± SD	CV (%)	95% Limit of Agreement
**TIME-DOMAIN**									
Mean RR (ms)	0.963	0.940, 0.977	20.56	2.54	56.99	7.05	1.01 ± 5.31	0.86	−9.40, 11.44
SDNN	0.996	0.993, 0.997	0.98	3.16	2.73	8.76	0.05 ± 1.63	2.47	−3.15, 3.26
Mean HR (bpm)	0.958	0.932, 0.974	2.18	2.88	6.04	7.98	−0.10 ± 0.53	0.86	−1.15, 0.95
RMSSD (ms)	0.998	0.997, 0.999	0.90	2.68	2.49	7.44	0.12 ± 1.50	2.29	−2.90, 3.10
RR Tri	0.989	0.983, 0.993	0.42	4.96	1.15	13.74	0.10 ± 0.79	3.97	−1.45, 1.65
**FREQUENCY DOMAIN**									
LF (n.u.)	0.971	0.953, 0.982	3.34	7.12	9.27	19.74	−0.93 ± 5.45	6.69	−11.60, 9.76
HF (n.u.)	0.970	0.952, 0.982	3.40	6.43	9.44	17.83	0.93 ± 5.45	6.02	−9.74, 11.60
LF (ms^2^)	0.931	0.889, 0.957	117.72	26.59	326.30	73.70	−22.54 ± 305.2	14.38	−620.70, 575.70
HF (ms^2^)	0.979	0.965, 0.987	110.31	17.47	305.76	48.43	−8.94 ± 191.10	9.48	−383.40, 365.60
**NONLINEAR METHODS**									
Apen	0.912	0.858, 0.945	0.20	18.54	0.56	51.39	−0.01 ± 0.03	1.45	−0.07, 0.07
SampEn	0.970	0.951, 0.981	0.04	2.50	0.12	6.92	0.01 ± 0.08	2.32	−0.14, 0.16
DFA α1	0.957	0.930, 0.973	0.05	5.71	0.15	15.81	−0.01 ± 0.08	5.93	−0.17, 0.17
DFA α2	0.937	0.899, 0.961	0.04	11.12	0.11	30.81	0.01 ± 0.05	8.83	−0.11, 0.11

HRV: Heart Rate Variability; RR: interbeat intervals between all successive heartbeats; SDNN: standard deviation of the N-N interval; RMSSD: root mean square differences of successive RR intervals; RR Tri: integral of the RR intervals histogram divided by the height of the histogram; TINN: baseline width of the RR intervals histogram; LF: normalized unit in the low-frequency band; HF: normalized unit in the high-frequency band; LF/HF: ratio of LF-to-HF; ApEn: approximate entropy; SampEn: sample entropy; DFA α1: detrended fluctuations analysis, which describes short-term fluctuations; DFA α2: detrended fluctuation analysis, which describes long-term fluctuations; ms: milliseconds; n.u.: normal units; ms: milliseconds.

## Data Availability

The datasets used and/or analyzed during the current study are available from the corresponding author on reasonable request.

## Supplementary Materials

The following supporting information can be downloaded at: https://www.mdpi.com/article/10.3390/ijerph192013587/s1. Bland-Altman plots for all variables analyzed in time domain, frequency domain and non-linear analysis of Heart Rate Variability evaluated intrarrater and interrater.
