# Supplementary material for: Intra- and Interrater Reliability of Short-Term Measurement of Heart Rate Variability on Rest in Individuals Post-COVID-19
Source: Int J Environ Res Public Health. 2022 Oct 20;19(20):13587. doi: 10.3390/ijerph192013587 (PMC9602575; doi:10.3390/ijerph192013587)

SUPPLEMENTARY MATERIAL

INTER- AND INTRA-RATER RELIABILITY OF SHORT-TERM MEASUREMENT OF HEART RATE VARIABILITY ON REST IN COVID-19.

Lucivalda Viegas de Almeida [1,2], Aldair Darlan Santos-de-Araújo [3,4], Rodrigo Costa Cutrim [2,5], Rudys Rodolfo de Jesus Tavarez [5], Audrey Borghi-Silva [3,4], Fábio Henrique Ferreira Pereira [2,6], André Pontes-Silva [3], Adriana Sousa Rêgo [1,2,6,8,9], Daniel Santos Rocha [2,8], Renan Shida Marinho [3,4], Almir Vieira Dibai-Filho [7,9] and Daniela Bassi-Dibai [1,2,5,6,8,*]

[1]Postgraduate Program in Programs Management and Health Services, Universidade Ceuma, São Luís, MA, Brazil
[2]Department of Physical Therapy, Universidade Federal de São Carlos, São Carlos, SP, Brazil
[3]Postgraduate Program in Dentistry, Universidade Ceuma, São Luís, MA, Brazil
[4]Postgraduate Program in Environment, Universidade Ceuma, São Luís, MA, Brazil
[5]Department of Physical Therapy, Universidade Ceuma, São Luís, MA, Brazil
[6]Postgraduate Program in Adult Health, Universidade Federal do Maranhão, São Luís, MA, Brazil
[7]Grupo de pesquisa em Avaliação e Reabilitação Cardiovascular,Respiratória e Metabólica, Universidade CEUMA, São Luís, MA, Brazil
[8]Cardiopulmonary Physiotherapy Laboratory – LACAP, Universidade Federal de São Carlos, São Carlos, SP, Brazil
[9]Grupo de Pesquisa em Reabilitação, Exercício e Movimento (REMOVI) Universidade Federal do Maranhão, São Luís, MA, Brazil

*Correspondence: danielabassifisio@gmail.com.

Corresponding author:
Daniela Bassi-Dibai. Universidade Ceuma, Postgraduate Program in Programs Management and Health Services, São Luís, MA, Brazil. Street Josué Montello, number 1, Jardim Renascença. Zip Code 65075-120, São Luís, MA, Brazil. Telephone: +559832144277. E-mail: danielabassifisio@gmail.com.

INTRARATER (RESEARCHER 01)

1.0) MEAN RR

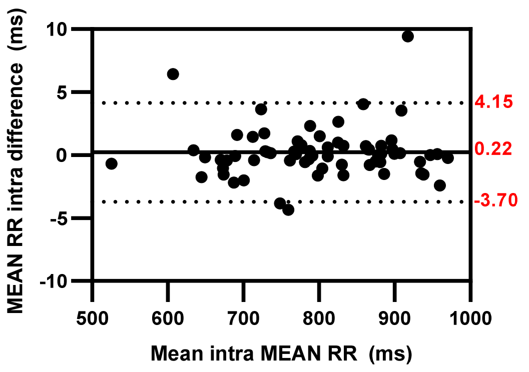

2.0) SDNN

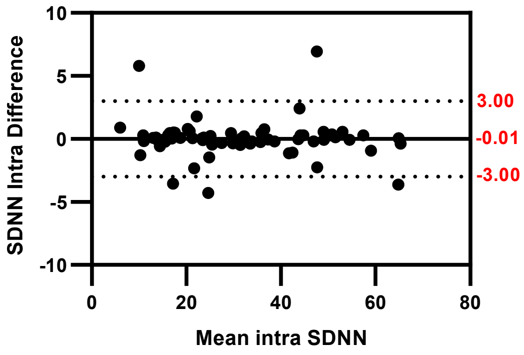

3.0) MEAN HR

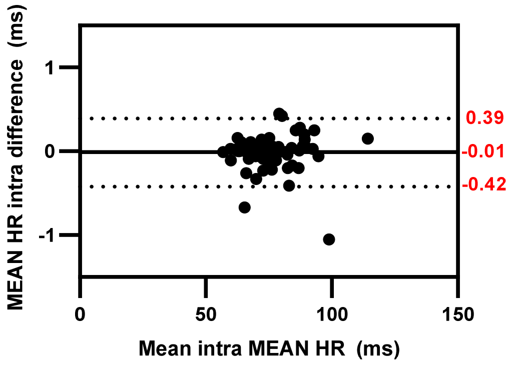

4.0) RMSSD

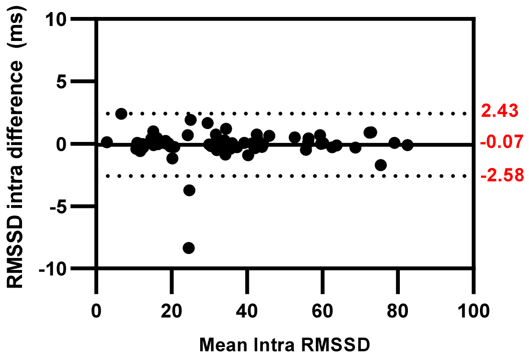

INTRARATER (RESEARCHER 02)

1.1) MEAN RR

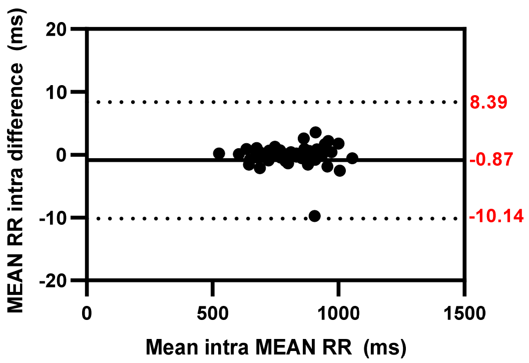

2.1) SDNN

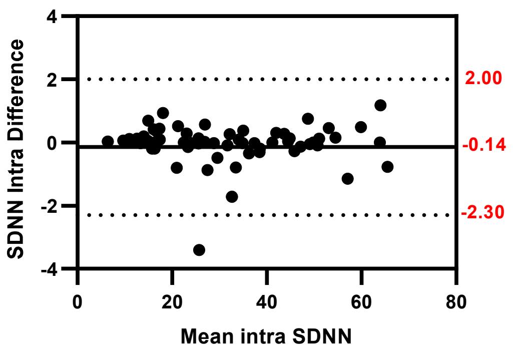

3.1) MEAN HR

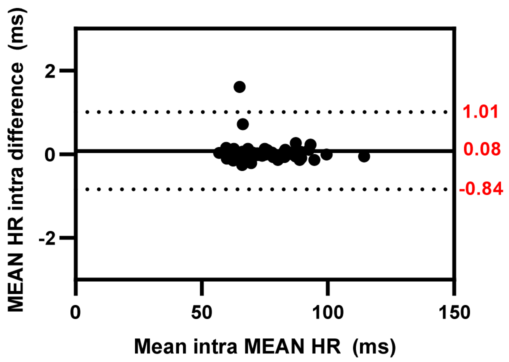

4.1) RMSSD

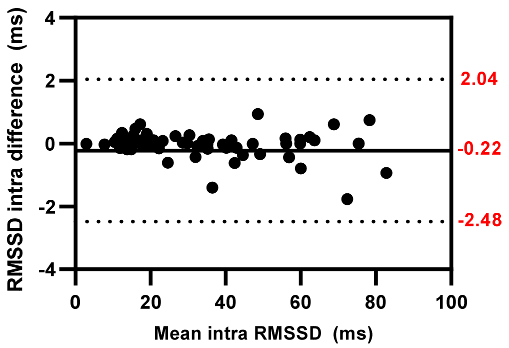

INTRARATER (RESEARCHER 01)

5.0) RR Tri

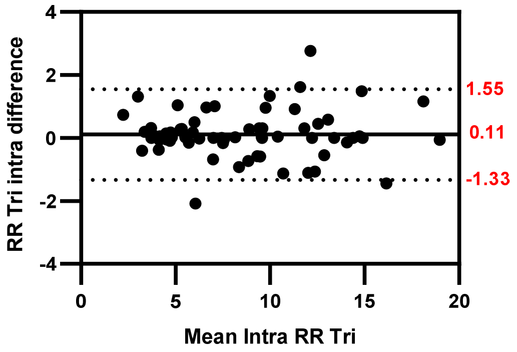

6.0) LF (n.u)

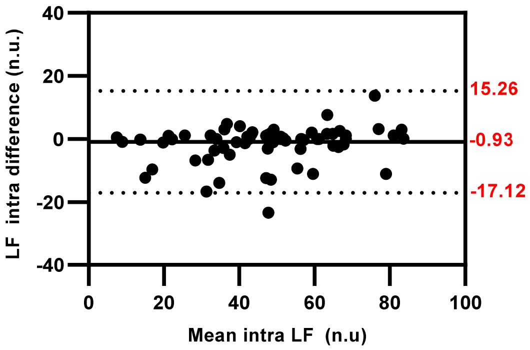

7.0) HF (n.u.)

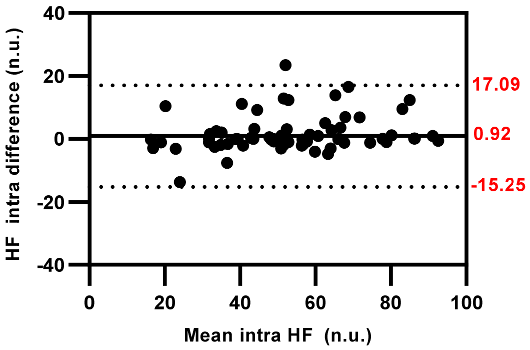

8.0) LF (ms²)

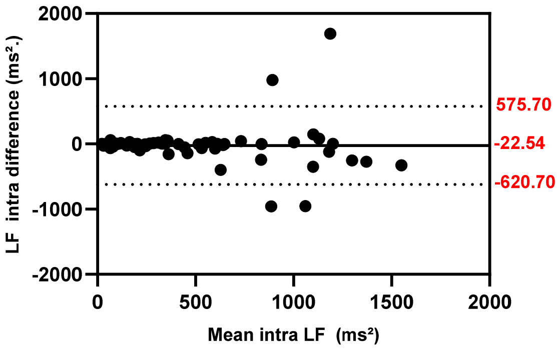

INTRARATER (RESEARCHER 02)

5.1) RR Tri

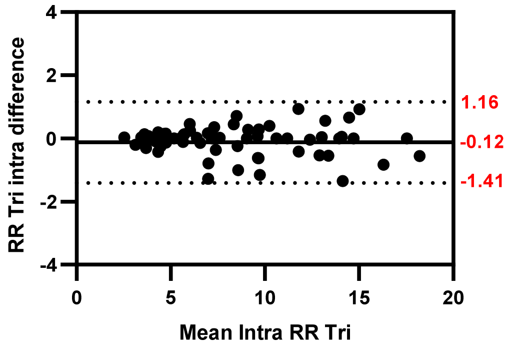

6.1) LF

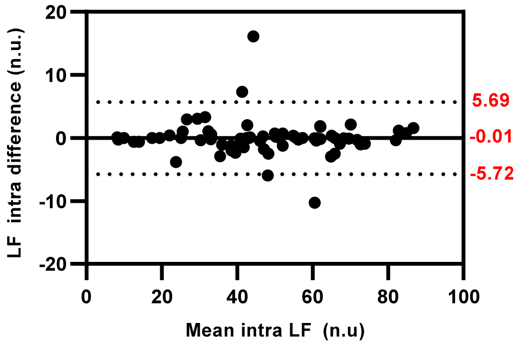

7.1) HF

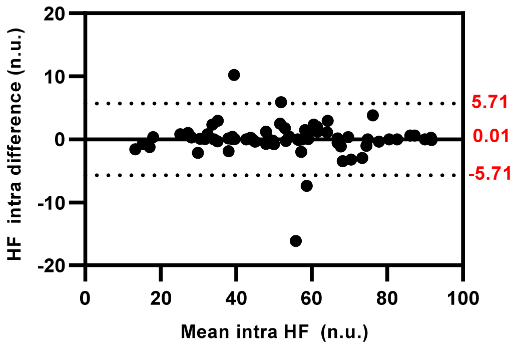

8.1) LF (ms²)

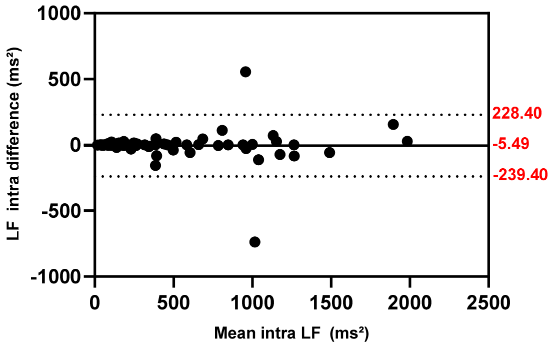

INTRARATER (RESEARCHER 01)

9.0) HF (ms²)

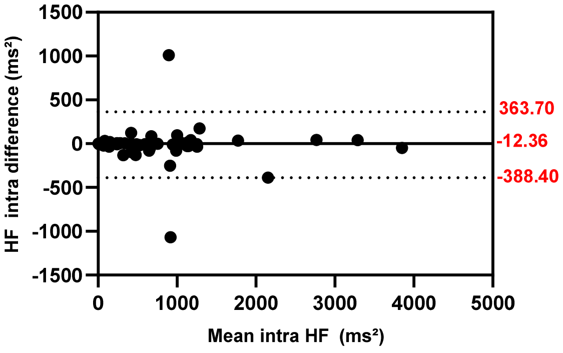

10.0) APEN

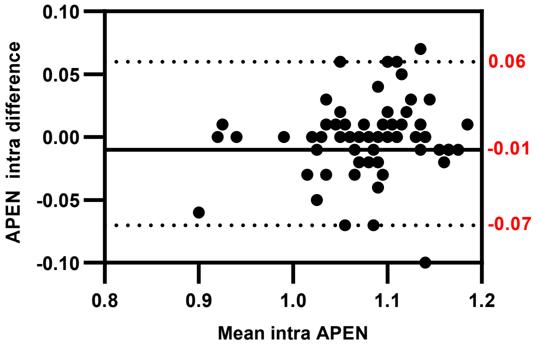

11.0) SAMPEN

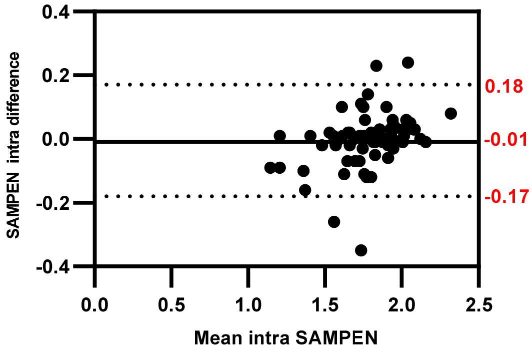

12.0) DFA α1

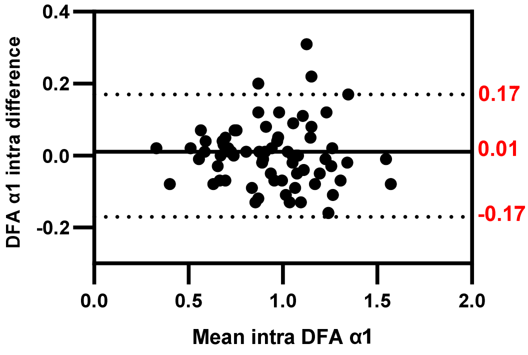

INTRARATER (RESEARCHER 02)

9.1) HF (ms²)

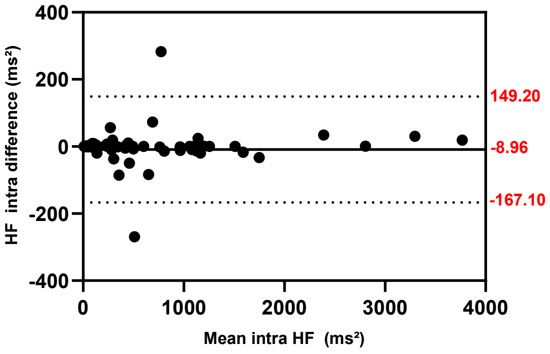

10.1) APEN

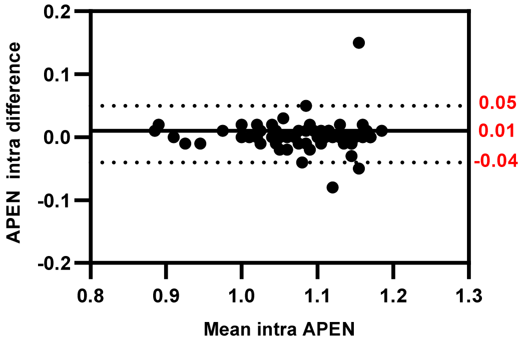

11.1) SAMPEN

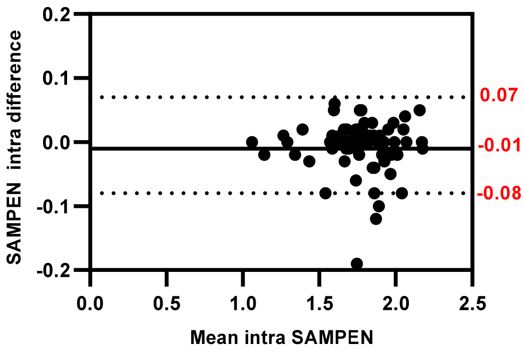

12.1) DFA α1

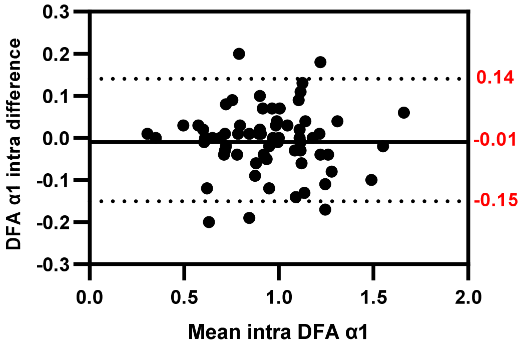

INTRARATER (RESEARCHER 01)

13.0) DFA $\alpha2$

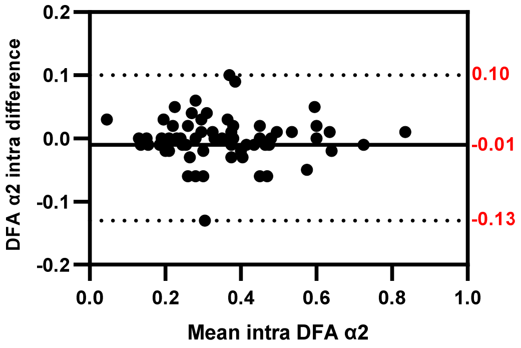

INTRARATER (RESEARCHER 02)

13.1) DFA $\alpha2$

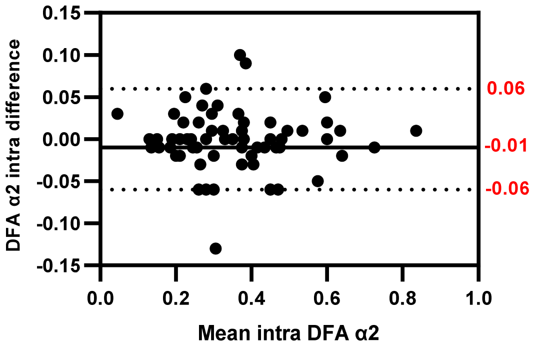

INTERRATER

1.0) MEAN RR

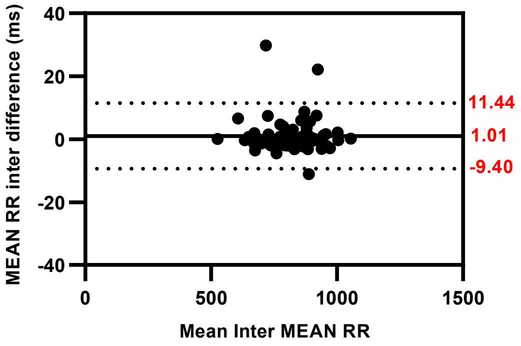

2.0) SDNN

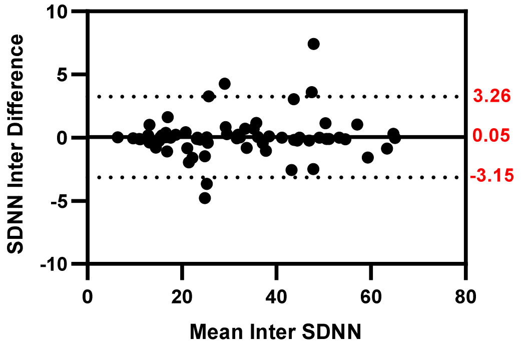

3.0) MEAN HR

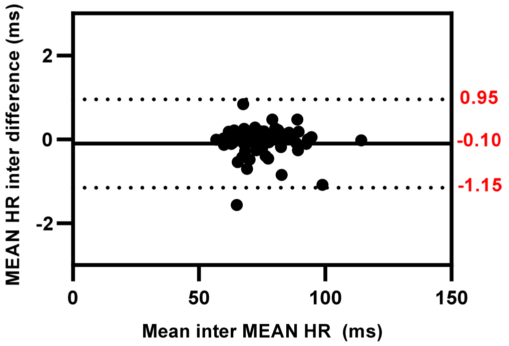

4.0) RMSSD

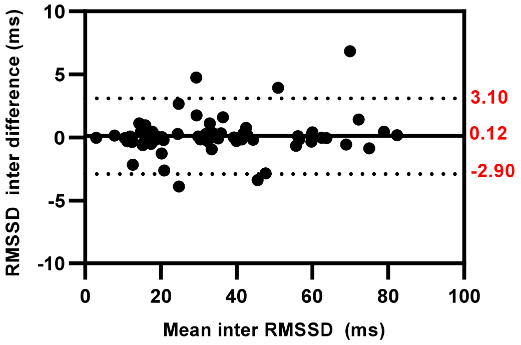

5.0) RR Tri

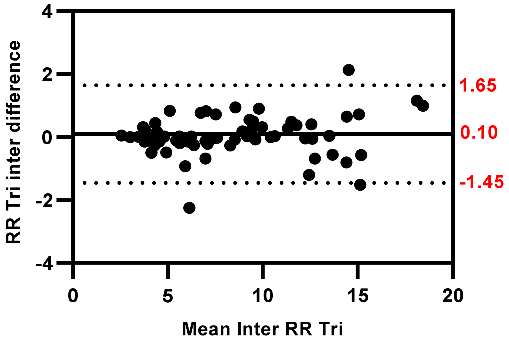

6.0) LF (n.u.)

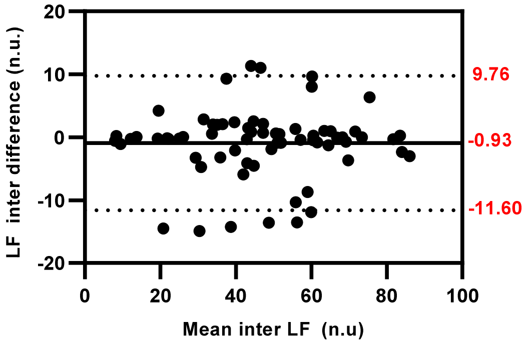

7.0) HF (n.u.)

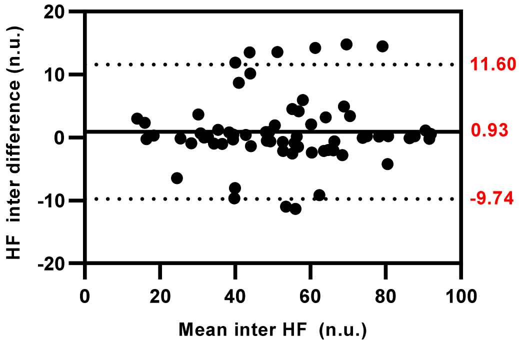

8.0) LF (ms²)

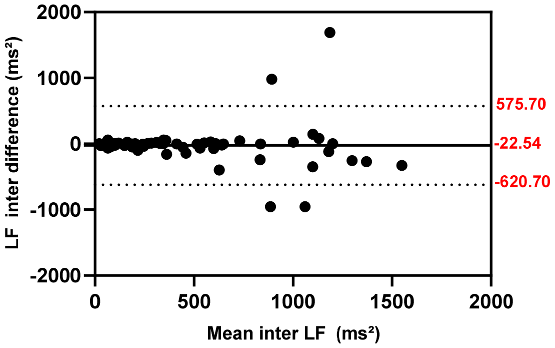

INTERRATER

9.0) HF (ms²)

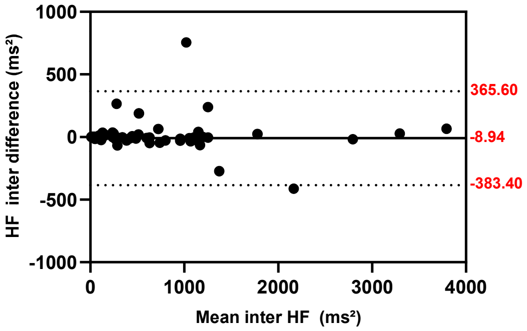

10.0) APEN

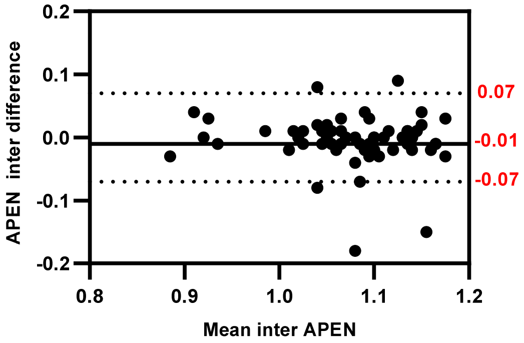

11.0) SAMPEN

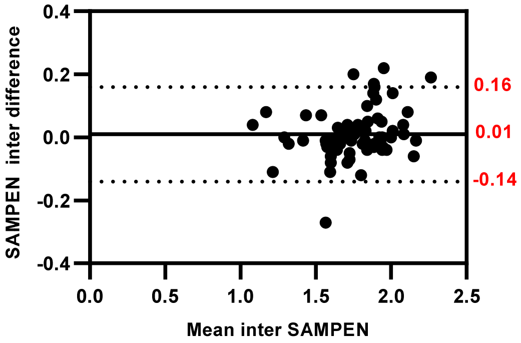

12.0) DFA $\alpha 1$

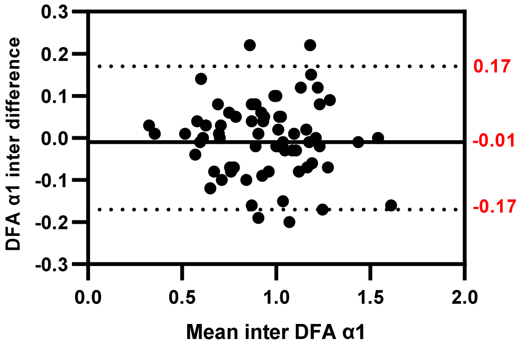

INTERRATER

13.0) DFA $\alpha 2$

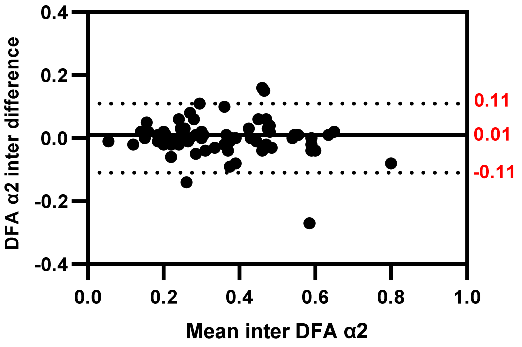

Supplement: Supplementary file 1 [file ijerph-19-13587-s001.zip › ijerph-1887538-supplementary/ijerph-1887538-supplementary.pdf]
